# Mechanism of Lower Airway Hyperresponsiveness Induced by Allergic Rhinitis

**DOI:** 10.1155/2022/4351345

**Published:** 2022-07-12

**Authors:** Yiting Liu, Jichao Sha, Cuida Meng, Dongdong Zhu

**Affiliations:** ^1^Department of Otolaryngology Head and Neck Surgery, China-Japan Union Hospital of Jilin University, Changchun, China; ^2^Jilin Provincial Key Laboratory of Precise Diagnosis and Treatment of Upper Airway Allergic Diseases, China

## Abstract

Allergic rhinitis is a global illness that puzzles many researchers. Most patients with allergic rhinitis also have lower airway hyperresponsiveness, and an allergic rhinitis attack can increase lower airway hyperresponsiveness. However, the mechanism of the effect of allergic rhinitis on the lower airways is still unclear. In this paper, the effects of allergic rhinitis on the lower airways are studied in terms of epidemiology, anatomy, pathophysiology, nasal function loss, inflammation drainage, nasobronchial reflex, and whole-body circulatory flow to determine the mechanism involved and provide ideas for future diagnosis, treatment, and experiments.

## 1. Introduction

Upper and lower airways are considered a unified morphological and functional unit, and the connection existing between them has been observed for many years, both in health and disease [1, 2]. The relationship between the upper airways, which consist of the two nasal passages and the paranasal sinuses, and the lower airways, which consist of the bronchi and bronchioles, is of great interest from both clinical and physiological standpoints [3, 4]. To understand illnesses that appear on the surface to affect only one element of an integral organ system, one should be able to understand and visualize the function of the entire system. The concept of a unified airway disease was suggested [5]. However, there is no clear conclusion about the effect of allergic rhinitis (AR) on the lower airways, especially on the hyperresponsiveness of the lower airways. This article will review the previous researches in this field to provide direction for future research on AR.

## 2. Epidemiology

AR currently affects approximately 40% of adults and 25% of children worldwide [6–8]. Several recently published studies have illustrated the connection of AR with asthma. This suggests that the evolution of asthma and rhinitis is bidirectional. Approximately 40% of AR patients have asthma symptoms, and approximately 80% of asthmatic patients have symptoms of AR [9–11]. In 1998, William et al. [12] reported a 23-year follow-up study that involved a total of 738 college students. The students' responses to the questionnaire revealed a frequent combination of asthma and AR. The results showed that 85.7% of patients with asthma had AR at the same time. Among patients with AR, asthma occurred in 21.3%. Another 23-year follow-up study conducted by Settipane et al. [13] included 1836 college students. The results demonstrated that two risk factors for developing new asthma were AR and positive allergy skin tests. Individuals with these two risk factors were approximately three times more likely to develop asthma than negative controls. Another 10-year follow-up study from Lombardi et al. [14] described a natural history of allergic disease evolution in 99 patients. One of their results showed that 31.8% of patients with AR developed concomitant asthma after 10 years.

## 3. Pathophysiology

The mucosa of the upper and lower airways is continuous, and they share many similar anatomical and histological characteristics [15]. The two parts of the respiratory mucosa, which is made up of pseudostratified ciliated columnar epithelium and the supporting lamina propria, have common structures, such as glands, ciliary epithelium, lamina propria, basement membrane, and goblet cells [16]. The epithelium is located above the basement membrane, which is mainly made up of type V laminin and type IV collagen. The function of the basement membrane is to separate the epithelium from the underlying mesenchymal components and make it possible for epithelial and inflammatory cells to migrate [17]. The lamina propria is under the basement membrane. The lamina propria contains serous glands, which are composed of water, polysaccharide, and collagen, and their function is to secrete mucus and keep the airway moist [18]. The ratio of extracellular matrix to serous glands plays a key role in regulating cell function, serving as the matrix and scaffold of various cell activities [19]. The epithelial tissue acts as a barrier by forming a continuous layer with almost no intracellular gaps, and it protects the body from environmental stress and physical damage. Additionally, airway epithelial cells not only comprise a physical barrier but also produce antimicrobial peptides, several chemokines, and cytokines which play key roles in immune, inflammatory, repair, and remodelling responses upon encounters with triggers, including inhaled allergens and pathogens [20].

The nasal mucosa contains many vessels, whereas the bronchial mucosa contains many smooth muscle cells [4, 21]. Under healthy conditions, epithelial cells can quickly repair when suffering from inflammatory injury. However, chronic epithelial damage causes structural changes in the airways [22], including epithelial shedding, goblet cell metaplasia, basement membrane thickening, extracellular matrix deposition in the submucosa, smooth muscle hypertrophy, subepithelial angiogenesis, and myofibroblast hyperplasia [23]. Defects in the epithelial barrier, the so-called leaky epithelium, have been demonstrated in affected organs in asthma, chronic rhinosinusitis, and allergic rhinitis, leading to the development of the epithelial barrier hypothesis in the pathogenesis of these diseases [24, 25]. Related experiments [26, 27] have reported the defects in the epithelial barrier of the nasal epithelium in patients with both seasonal and perennial allergic rhinitis (PAR).

Allergen immunotherapy (AIT) is a curative treatment for allergic diseases, such as AR and asthma, that alters the underlying pathophysiology, alters the skewed Th2 response, and induces long-term clinical tolerance against allergens [28, 29]. AIT leads to a significant reduction in symptoms and medication use, improves quality of life, and prevents the development of new sensitizations [30]. Furthermore, AIT prevents the development of asthma in patients with allergic rhinitis and the progression of prevalent asthma [31].

## 4. The Effect of Nasal Dysfunction on the Lower Airways

The loss of nasal function due to mucosal congestion and retention in the nasal cavity has a significant effect on the lower airways. In allergic diseases, complete or partial obstruction of the nose prevents airflow through the nasal cavity, and oral respiration becomes obligatory. Shturman-Ellstein et al. have shown that oral breathing associated with nasal obstruction will increase the intensity of bronchospasms [32].

The main physiological function of the nose is to heat and humidify the air inhaled before airflow reaches the lower airways. Resistance to airflow is less in oral breathing than in nasal breathing, but the air will be cooler and drier. These changes in air may affect the degree of bronchoconstriction. Proctor [33] concluded that at an ambient temperature of 23°C, air temperature increases to approximately 30°C in the midnasal passage, approximately 33°C in the nasopharynx, and only slightly above that in the upper trachea. At each of these sites, the relative humidity is nearly 100%. The subepithelial capillary network of the nasal cavity and the venous sinus below it accumulate a large amount of blood, leading to submucosal tissue hyperaemia and increased contact of the surface with airflow [33, 34], which is conducive to the distribution of heat and water transport. Therefore, these structures contribute to the humidification and heating of nasal air. Griffin et al. [35] carried out an experiment in which asymptomatic asthmatic and healthy control subjects inhaled cold air at equal ventilation through their noses or mouths in a random fashion, and the temperature in the retrotracheal oesophagus and pulmonary mechanics were recorded before and after ventilation. A linear relationship between the degree of airway cooling and the severity of subsequent bronchoconstriction was found in asymptomatic asthmatic patients. The healthy control subjects showed similar changes in temperature, but their lung function did not change. Assanasen et al. [36] conducted a prospective, parallel experiment on healthy control subjects, patients with seasonal AR and perennial AR, and patients with asthma. The temperature and humidity of the air were measured when cold, dry air entered and left the nasal cavity. The results showed that the noses of patients with asthma had a reduced ability to condition cold, dry air compared with those of healthy control subjects but a similar ability to those of seasonal AR subjects out of season.

Immunological function is another important function of the nose and includes the activity of nonspecific and specific systems. Nonspecific systems filter the particles and gaseous materials in inhaled air before they reach the lower airways [37]. The nasal mucus and mucocilia play an essential role in this process. The nasal mucus carries surface secretions backward to the nasopharynx through the sweeping action of the nasal mucocilia, which are located from the anterior nasal cavity to the nasopharynx. These materials are dispatched to the stomach periodically through swallowing and destroyed by gastric enzymes [38]. At the most anterior portion of the inferior turbinates, the cilia propel the mucus anteriorly towards the nasal vestibule. This action enables the clearance of deposited foreign materials by nose blowing, wiping, and so on, limiting true access of these foreign particles to the body [39]. In addition, the presence of these foreign materials will lead to the release of several factors through the activation of inflammatory monocytes, and macrophages will be mobilized to eliminate viral and cellular debris. The submucosal glands release antifactors, such as lysozyme and lactoferrin [40], or chemical defences, such as uric acid, in the epithelial lining fluid [41]. Specific immune functions facilitate the coupling of IgA with the secretory component-specific defence system, which consists of immunological humoral and cellular responses. This system is responsible for the complete elimination of pathogens and for the induction of immunological memory phenomena [41–43].

Another theory is that the blockade of the paranasal sinus and the loss of nasal epithelial function may reduce the release of nitric oxide (NO) in the airways. NO plays an important protective role in the upper airway. Lundberg et al. [44, 45] demonstrated that there is a large amount of NO in the upper airway, mainly from the nasal sinus. Other experiments have shown that NO has strong antiviral and antibacterial activities, improves oxygenation, plays a role in bronchodilation, and regulates the reactivity of the lower airways [46–48].

Complete or partial obstruction of the nose leads to air flowing directly into the lower airways without passing through the nasal cavity, and allergens or cold air is inhaled directly into the bronchus, causing hyperresponsiveness of the lower airways.

## 5. Upper Airway Drainage of Inflammatory Mediators

Upper airway drainage of inflammatory mediators is another mechanism by which AR leads to lower airway hyperresponsiveness. The nasopharynx, oropharynx, laryngopharynx, and lower airways are interconnected areas, and allergens that are inhaled into the upper airway may be discharged into the lower airways. Brugman et al. [49] performed animal experiments to measure bronchial reactivity when histamine was inhaled. The responsiveness to histamine increased with the presence of upper airway inflammation, and this responsiveness could be prevented when the draining of exudate out of the larynx was blocked. The results of this experiment demonstrated that nasal drainage can increase bronchial responsiveness. Huxley et al. [50] documented pulmonary aspiration among both healthy patients and patients with depressed consciousness with a radiolabelled marker that was released into the lower airways. Sato [51] used white-pepper-irritated nasal mucosa to examine airway resistance in laryngectomized patients and healthy adults, and they found that airway resistance increased after nasal mucosa irritation in healthy adults but that there was no such change in laryngectomized patients. The anatomical changes in laryngectomized patients may have led to the nose being unable to drain the increased secretions following white pepper stimulation into the lower airways. However, Bardin et al. [52] found that the drainage of secretions into the lower airways is unlikely to lead to coexistent pulmonary disease. They investigated pulmonary aspiration in a study by means of a technique in which a radionuclide was placed in a maxillary sinus. After 24 hours, no radionuclide was found in the lungs of any patient, but the radionuclide was found in the maxillary sinus nasopharynx, oesophagus, and lower gastrointestinal tract. The authors pointed out that the association was probably related to generalized mucosal disease affecting both the upper and lower airways.

## 6. The Nasobronchial Reflex

In 1919, Sluder pointed out that asthma may be caused by neural reflexes of the nose. The nasobronchial reflex is a branch of the diving reflex, which leads to the suppression of respiration, laryngospasm, and bronchoconstriction when the head is immersed in water [53]. This reflex leads to an increase in airway resistance caused by bronchoconstriction [54]. The smooth muscle contraction of the bronchus is caused by the efferent pathway of the vagus nerve, which originates from the sensory nerve endings in the nose that enter the central nervous system through the trigeminal nerve [55]. Because of the changes mentioned above, mammals can remove water from their lungs when diving. However, when harmful substances are inhaled in the nose (or when allergic patients inhale allergens), this reflex may induce immediate bronchoconstriction with the cessation of respiration in the expiratory phase due to the relaxation of inspiratory muscles.

This hypothesis underwent rigorous testing through a variety of animal experiments. Whicker and Kern [56] found that pulmonary resistance increased in awake and anaesthetized dogs after stimulation of the nasal mucosa. Whicker et al. [57] found that nasal stimulation typically produced marked changes in breathing patterns and a large transient increase in pulmonary airflow resistance. This response to nasal stimulation could be abolished by interrupting either the trigeminal nerve or the vagus nerve. In 1969, Kaufman et al. [58] measured tracheobronchial airway resistance by unilateral nose irritation in 5 patients who had undergone complete resection of the second division of the trigeminal nerve. There was no increase in resistance after irritation of the nose and nasopharynx on the resected side but a significant increase in resistance following stimulation of the intact side. In another experiment [59], they also showed that significant elevations in airway resistance were prevented by prior subcutaneous injections of atropine. Therefore, it can be concluded that the nasobronchial reflex results in bronchoconstriction by vagal and trigeminal pathways and the presence of afferent receptor sites in the nose. Other experiments demonstrated that allergen antigen exposure in AR patients increased the level of hyperresponsiveness in the lower airways [60]. Therefore, it can be concluded that the nasobronchial reflex results in bronchoconstriction by vagal and trigeminal pathways and the presence of afferent receptor sites in the nose.

Despite being well documented in those experiments, the existence of this reflex is still controversial [4]. Nadel and Widdicombe [61] found that there was no significant change in lung resistance after irritation of the nasal mucosa in decerebrated cats and anaesthetized cats. Schumacher et al. [62] reported a clinical trial of nasal stimulation with saline, allergen, and histamine in patients with asthma and control subjects and found that nasal symptomatology occurred without increases in lower airway resistance. Small et al. [63] measured the pulmonary function of patients with PAR 30 minutes after nasal challenge and found no significant decrease in flow rates. In addition, other experiments [64–67] demonstrated that inflammation will cause some changes in airway nerves, including an increase in nerve density and the excitability of airway nerves. Furthermore, the sensory nerves innervating the tissue produce more neuropeptides after the stimulation of inflammation [68]. The nasobronchial reflex may be strengthened by neural hyperresponsiveness caused by exogenous stimulated factors or endogenous immune factors [69]. The interaction between the immune and nervous systems will make the neural mechanism more complicated.

## 7. The Propagation of Inflammation via the Systemic Circulation

An allergic inflammatory response, such as increased eosinophil numbers deep in the nasal mucosa and then peripheral blood eosinophilia or other systemic inflammatory events, can be found in patients with AR after nasal allergen provocation. Eosinophils can selectively accumulate in the circulation and various tissues of patients with allergic diseases. Because eosinophils are potent proinflammatory effector cells, they have a role in defending parasites. However, the overaccumulation of eosinophils, which always occurs in an allergic inflammatory response, will cause severe host tissue damage [70]. In the lower airways, when stimulated, eosinophils can release some toxic proteins, including eosinophil cationic protein (ECP), eosinophil peroxidase (EPO), and major basic protein (MBP), which will damage the integrity of the epithelium of the lower airways, leading to a decrease in ciliated and brush cells, mast cell secretion, and the exposure of underlying sensory nerve endings, which promote bronchial hyperresponsiveness and bronchoconstriction [71–76].

**Figure 1:**
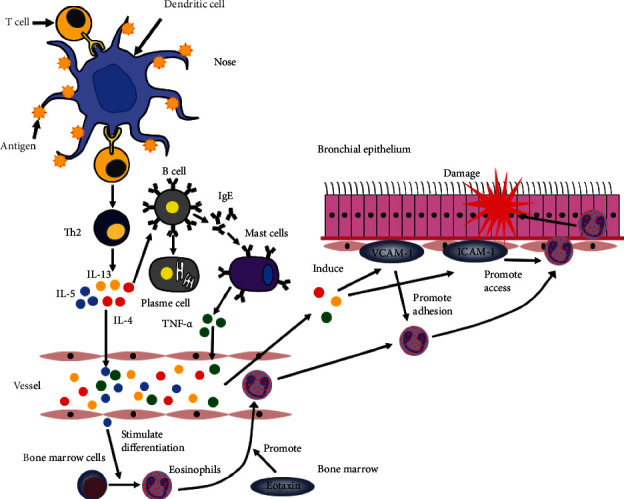
After the upper airway is stimulated by allergens, the inflammatory factors and cytokines produced by the upper airway cause the aggregation of eosinophils in the lower airway, which causes the hyperresponsiveness of the lower airway.

The allergic reaction of AR starts with the recognition of allergens by antigen-presenting cells, mainly dendritic cells, which drive type 2 T helper (Th2) cell polarization. Then, Th2 cells release various inflammatory cytokines, including IL-4, IL-5, and IL-13, leading to the recruitment of effector cells, such as eosinophils and mast cells [77, 78]. These inflammatory factors and eotaxin have an important function in the generation and recruitment of effector cells, such as eosinophils, in the lower airways. Experiments have reported higher concentrations of eotaxin in the epithelium of the nose and bronchus (mainly in macrophages) in patients with allergic inflammation [79, 80]. The absorption of IL-5 from the nose into the blood after allergen challenge can mediate the increase in the differentiation of progenitors into eosinophils in bone marrow [81]. Eotaxin is a chemokine that accelerates the egress of eosinophils from bone marrow, which has a significant synergism with IL-5 mobilization of mature eosinophils from the femoral marrow [82]. Then, IL-4 and IL-13 upregulate vascular cell adhesion molecule-1 (VCAM-1) on the endothelium. Eosinophils become tethered to the endothelium through the effects of VCAM-1 and then transfer into the endothelium through leukocyte function-associated antigen-1 (LFA-1) and intercellular adhesion molecule-1 (ICAM-1) [81]. These inflammatory factors and adhesion molecules, including TNF-*α*, which is stored in mast cells and released upon IgE-dependent stimulation, are involved in this process [83–85] (Figure 1).

Palframan et al. [82] found that the injection of eotaxin into guinea pigs stimulated an increase in blood eosinophilia and a corresponding decrease in the number of eosinophils retained in the bone marrow. The preincubation of bone marrow cells with IL-5 produced considerably more eosinophils than preincubation with eotaxin. Braunstahl et al. [86] found that at 24 hours after nasal allergen provocation, an influx of eosinophils and increased expression of vessels positive for ICAM-1, VCAM-1, and e-selectin vessels were detected in the nasal epithelium and its lamina propria, as well as in the bronchial epithelium and its lamina propria. The number of mucosal eosinophils correlated with the expression of ICAM-1, e-selectin, and VCAM-1 in patients with AR. Sedgwick et al. [87] observed bronchoalveolar lavage fluid obtained from AR patients and found a significant increase in histamine and tryptase 12 minutes after challenge with antigen and another increase in IL-5 concentrations that correlated with the presence of eosinophils and eosinophil granular proteins 48 hours after challenge. Neither eosinophils nor soluble mediators of eosinophils were increased in healthy control subjects.

## 8. Summary

The influence of AR on the lower airways is very complex and cannot be explained by only one of the above mechanisms but may be the common result of the interaction of several mechanisms. Within a short time after AR, the hyperresponsiveness of the lower airways may be caused by the rhinopulmonary reflex, lower airway drainage of allergens, and nasal obstruction. From the verification experiments for various mechanisms, we noticed that in the rhinopulmonary reflex test, the time of AR-induced lower airway hyperresponsiveness was short because sensory nerves of the nose can quickly convert the stimulation into an electrical signal, which can be quickly transmitted to the lower airways through A-fibres and produce corresponding complications [68]. In addition, allergens directly drained from the nose or untreated air can quickly reach the lower airways through the normal anatomical structure. Therefore, the above three mechanisms may provide a reasonable explanation for the occurrence of lower airway hyperresponsiveness within a short time after AR. However, after prolonged nasal stimulation, lower airway hyperresponsiveness is more caused by circulating inflammatory factors because it takes a certain time for IL-5 to stimulate bone marrow cells to differentiate into eosinophils and for IL-4 and IL-13 to upregulate adhesion- and chemotaxis-related proteins.

The evidence for these complex, multiple mechanisms also provides some inspiration for follow-up experiments. Until now, the mechanism of propagation of inflammation via the systemic circulation has been a common view, and evidence for several other mechanisms remains controversial. In addition to some defects in the experiments themselves, evidence for the existence of several mechanisms shows that experimental manipulation of these mechanisms individually can lead to interference with other mechanisms and thus incorrect results. Therefore, in future experiments, we can target these mechanisms individually in both animal and human experiments to avoid affecting several mechanisms. For example, in a mouse experiment, the trigeminal nerve of the mouse can be cut off in advance to block the influence of the nasobronchial reflex. When nasal irritation is administered, allergens can be administered in a way that avoids the inhalation of allergens into the lower airways. For example, cotton pledgets with allergens in inferior turbinates or head-down positions and tracheal cannulas have been used in animal experiments [49, 88]. These methods can reduce the impact of allergens directly drainage and inhalation into the lower airways.

In conclusion, the mechanisms of AR-induced lower airway hyperresponsiveness are complex. This review provides some exploration of the details. At the same time, this review also forward some noteworthy features for future experiments to avoid the interactions among several mechanisms and to conduct more accurate experiments.
